# Special Issue of Pathogenesis of Pregnancy-Related Complications, 2023

**DOI:** 10.3390/ijms25052487

**Published:** 2024-02-20

**Authors:** Ilona Hromadnikova

**Affiliations:** Department of Molecular Biology and Cell Pathology, Third Faculty of Medicine, Charles University, 100 00 Prague, Czech Republic; ilona.hromadnikova@lf3.cuni.cz; Tel.: +420-296511336

This Special Issue mainly focuses on preeclampsia (PE), haemolysis, elevated liver enzymes, and low platelet count (HELLP) syndrome, gestational diabetes mellitus (GDM), foetal growth restriction (FGR), small-for-gestational-age foetuses (SGA), miscarriage, stillbirth, first-episode psychosis (FEP) during pregnancy, and pregnancy-related acute kidney injury (PR-AKI). It provides an overview of the latest research on pathophysiological mechanisms in these pregnancy-related complications and introduces novel modalities to predict the onset of late miscarriage, stillbirth, or HELLP syndrome, within the framework of the first trimester screening programme.

In addition, special attention is paid to immune mechanisms induced by rubella virus (RuV) infection in trophoblasts. Moreover, in a case report, a newborn diagnosed with Beckwith–Wiedemann Syndrome (BWS), an imprinting disorder, born to a mother with a history of PE and HELLP syndrome is discussed.

This Special Issue (closed on 31 December 2023) contains three reviews, six original papers, and one case report.

In their review, Hu and Zhang [1] summarize the role of Ca^2+^-activated K^+^ channels (K_Ca_) in the regulation of uteroplacental circulation in normal gestation and pregnancies complicated by preeclampsia, foetal growth restriction, and gestational diabetes. K_ca_ channels have been reported to participate in the setting and regulation of the resting membrane potential of vascular smooth muscle cells and endothelial cells. In addition, K_ca_ channels have been shown to control vascular tone and blood pressure.

Mora-Palazuelos et al. [2] present a review in which they conducted a survey on the role of non-coding RNAs (ncRNAs) in immune dysregulation present in preeclampsia. This review summarizes the role of microRNAs and long non-coding RNAs (lncRNAs) in multiple processes such as type 1 immune response regulation; trophoblast cell proliferation, migration, and promotion of invasion and autophagy; placental development and angiogenesis; and the immune microenvironment regulation in placenta promoting inflammatory factors and the population of M1 and M2 macrophages.

Kornacki et al. [3] review the pathophysiology of preeclampsia. They explain that next to an impaired placental perfusion, which is a crucial phenomenon of the disease, heart dysfunction should be considered as the primary cause of preeclampsia. This more complex approach might lead to more personalized and precise care of patients with preeclampsia.

Giommi et al. [4] describe the changes in placentas derived from patients with gestational diabetes mellitus (GDM) and small-for-gestational-age foetuses (SGA). Increased levels of superoxide dismutase 1 (SOD-1) and catalase (CAT), which correlated with hyperglycaemia, were observed in GDM patients. Moreover, GDM and SGA altered collagen structures and modified the lipidic composition of placental chorionic villi. In addition, SGA decreased collagen deposition.

Our research group [5] demonstrates a novel option for how to predict the later occurrence of HELLP syndrome during the first trimester of gestation based on the altered expression of microRNA biomarkers in maternal whole peripheral blood and selected maternal clinical characteristics that represent risk factors for the development of HELLP syndrome.

Schultz et al. [6] report their study of the response of trophoblasts to rubella virus (RuV). They demonstrated that, in BeWo cells, RuV infection induced type III interferon (IFN) production. In addition, they observed the increased production of type I INF-β and type III IFNs when they transfected trophoblast cells with dsRNA analogue. INF-β and type III IFN-λ1 inhibited RuV.

Our research group [7] also introduces a first-trimester predictive model for late miscarriage and stillbirth based on the altered expression of microRNA biomarkers in maternal whole peripheral blood and selected maternal clinical characteristics that represent risk factors for miscarriage and stillbirth.

Ortega et al. [8] present their analysis of gene and protein expression in placental tissues of women after first-episode psychosis (FEP) during pregnancy. They observed increased gene and protein expression of OXT, AVP, OXTR, and AVPR1A and suggested that FEP during pregnancy might lead to abnormalities in the paracrine and endocrine activity of the placenta.

Griffin et al. [9] detail their research, in which administration of indoxyl sulphate (I.S), a uremic toxin, in a Sprague Dawley rat during pregnancy elicited renal injury comparable to pregnancy-related acute kidney injury (PR-AKI), which is induced in animals by bilateral renal ischemic reperfusion surgery. They also demonstrated that administration of I.S led to an increased blood–brain barrier permeability.

Staniczek et al. [10] discuss, in a case report, a newborn diagnosed with Beckwith–Wiedemann Syndrome (BWS), an imprinting disorder, born to a mother with a history of PE and HELLP syndrome. In this particular case, FGR atypically occurred in the newborn, and no mutations in the *CDKN1C* gene, commonly associated with BWS, were present.
